# Graphene-enabled electron microscopy and correlated super-resolution microscopy of wet cells

**DOI:** 10.1038/ncomms8384

**Published:** 2015-06-11

**Authors:** Michal Wojcik, Margaret Hauser, Wan Li, Seonah Moon, Ke Xu

**Affiliations:** 1 Department of Chemistry, University of California, Berkeley, California 94720, USA; 2 Life Sciences Division, Lawrence Berkeley National Laboratory, Berkeley, California 94720, USA

## Abstract

The application of electron microscopy to hydrated biological samples has been limited by high-vacuum operating conditions. Traditional methods utilize harsh and laborious sample dehydration procedures, often leading to structural artefacts and creating difficulties for correlating results with high-resolution fluorescence microscopy. Here, we utilize graphene, a single-atom-thick carbon meshwork, as the thinnest possible impermeable and conductive membrane to protect animal cells from vacuum, thus enabling high-resolution electron microscopy of wet and untreated whole cells with exceptional ease. Our approach further allows for facile correlative super-resolution and electron microscopy of wet cells directly on the culturing substrate. In particular, individual cytoskeletal actin filaments are resolved in hydrated samples through electron microscopy and well correlated with super-resolution results.

## Abstract (web summary)

Preparing biological material for electron microscopy (EM) involves harsh processing steps that can poorly preserve cellular ultrastructure. Here the authors apply a single layer of graphene onto wet cells to enable direct EM using low voltage, and correlate actin filaments and mitochondria using super-resolution microscopy.

## Graphical abstract

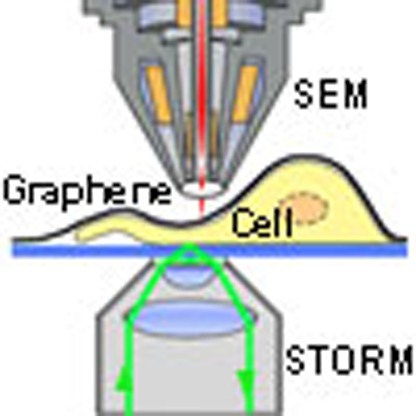

A major challenge in the application of electron microscopy to biological samples has been faithful preservation of cellular ultrastructure during the laborious dehydration and embedding/coating procedures required for sample preparation^1^,^2^,^3^. The harsh procedures are also detrimental to fluorescence^4^, thus introducing difficulties for correlating structural electron microscopy information with molecular specificity from high-resolution fluorescence microscopy, including super-resolution methods^4^,^5^,^6^,^7^. Quick freezing, as performed in cryo-electron microscopy methods, circumvents the need for dehydration^8^,^9^, but requires dedicated equipment and is challenging for whole animal cells. Micro-fabricated liquid enclosures enable direct electron microscopy of hydrated cells^9^,^10^,^11^,^12^,^13^,^14^, but such devices are difficult to fabricate, and the relatively thick (>∼50 nm) suspended viewing windows employed often limit the obtainable contrast and resolution. Furthermore, the special substrates used in cryo-electron microscope and liquid enclosures are difficult to adapt to oil-immersion lenses^14^ for correlation with high-resolution optical microscopy methods.

Here we utilize graphene, a single-atom-thick honeycomb lattice of carbon atoms^15^, as an impermeable and conductive membrane to uniquely enable electron microscopy and correlated super-resolution microscopy of wet and untreated, or fixed mammalian cells cultured on conventional coverglass with exceptional ease. Despite being at the ultimate limit of membrane thinness, graphene is impermeable to gas and liquid^16^,^17^,^18^,^19^, electrically and thermally conductive^15^, and chemically inert. We previously reported the use of graphene for sealing surface-adsorbed molecules to interrogate their nano-structures with atomic force microscopy^20^,^21^, and noted that graphene can seal nanoscale water droplets in ultra-high vacuum^22^. Other studies showed that graphene serves as an excellent transparent support film for electron microscopy^23^,^24^, and can be used to entrap nanometre-scaled liquid to allow for electron microscopy of nanocrystals and protein in liquid^25^,^26^,^27^. Electron microscopy of multilayer graphene oxide-wrapped bacteria has been achieved via mixing of liquid suspensions of bacteria and micrometre-sized graphene oxide flakes^19^,^28^, but such approaches are difficult to apply to the much larger animal cells, and the sharp edges of graphenic flakes tend to penetrate the cell membrane and lead to internalization^29^.

We report that monolayer graphene can hermetically seal and protect large areas of mammalian cells, cultured on conventional coverglass, from external environments, including the high vacuum typically encountered in an electron microscope. This protection, combined with the high electrical and thermal conductivity of graphene and its ultimate thinness, enables facile electron microscopy of wet and untreated cells with excellent contrast and resolution, as well as correlated super-resolution microscopy directly on the culturing substrate. In particular, individual actin filaments are resolved in wet cells through electron microscopy and well correlated with super-resolution results.

## Results

### Graphene insulates cells from the external environment

**Figure 1:**
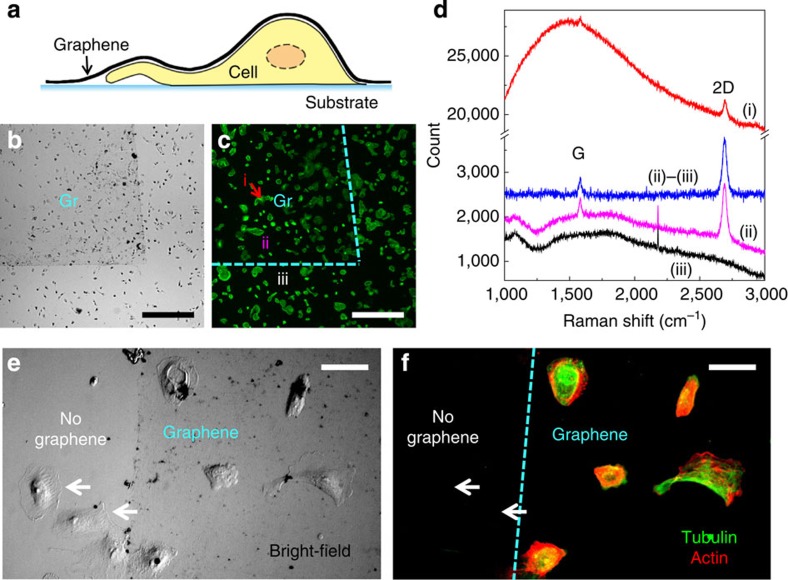
Graphene insulates cells from the external environment. (**a**) Schematic of our approach. (**b**,**c**) Graphene covering a region (Gr) of Alexa Fluor 488-phalloidin-labelled BS-C-1 cells on coverglass. (**b**) Bright-field microscopy. (**c**) Fluorescence microscopy of Alexa Fluor 488-phalloidin. (**d**) Raman spectroscopy for different areas of the sample: graphene on top of cell (i), graphene off cell (ii) and substrate not covered by graphene (iii). (ii)–(iii) denotes spectrum (ii) after subtraction of spectrum (iii). (**e**,**f**) Graphene-covered (right 2/3) and non-covered (left 1/3) labelled (green: Alexa Fluor 647-labelled tubulin; red: Alexa Fluor 555-labelled actin) BS-C-1 cells, after exposure to a sodium borohydride bleaching solution. (**e**) Bright-field image. (**f**) Fluorescence image of the labelled tubulin (green) and actin (red). Scale bars, 0.5 mm (**b**,**c**); 50 μm (**e**,**f**).

Graphene was produced by chemical vapour deposition (CVD) growth on copper foil and wet-transferred to cover large (∼10 × 10 mm^2^) areas of cells conventionally cultured on coverglass (Fig. 1a). Commercially available and homegrown graphene performed similarly in our experiments. Deposited graphene was identified in bright-field microscopy as a continuous, slightly darkened film (Fig. 1b). Meanwhile, no noticeable impact is observed for the labelled fluorescence in cells (Fig. 1c). Raman spectroscopy confirmed that the deposited graphene was a high-quality monolayer (Fig. 1d and Methods). The spectrum on graphene-covered cells had high background because of the labelled fluorescence in cells, but the 2D and G peaks of graphene^30^ are nonetheless clearly resolved (Fig. 1d).

To evaluate whether the monolayer graphene membrane can satisfactorily insulate cells from the external environment, fluorescently labelled cells were covered with graphene and then immersed in 0.1% sodium borohydride, a reducing agent commonly used to bleach fluorescence in biological samples, for 60 s (Fig. 1e,f). Cells not covered by graphene were bleached (for example, white arrows), whereas cells protected by graphene retained fluorescence. This result indicates that graphene provided a hermetic seal for cells. Long-term (16 h) insulating capability was further confirmed through dye labelling experiments (Supplementary Fig. 1).

### Graphene enables electron microscopy of wet cells

**Figure 2:**
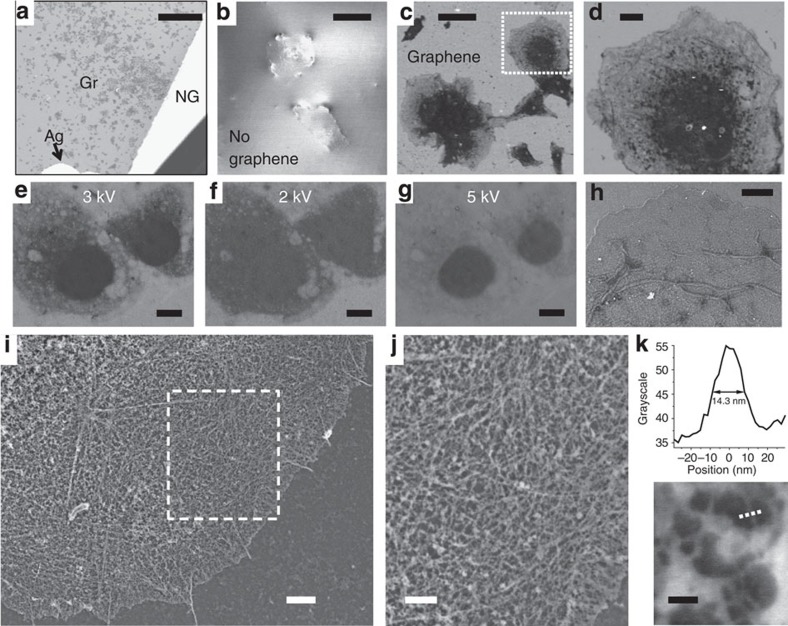
Graphene-enabled electron microscopy of wet cells. (**a**) Zoom-out SEM image of graphene-covered (Gr) and non-covered (NG), fixed and lightly stained wet COS-7 cells on coverglass. (**b**) Non-covered cells at higher magnification (*V*_0_=2 kV). (**c**) Graphene-covered cells in the same sample, image taken under the same conditions as **b**. (**d**) Zoom in of **c**. (**e**–**g**) SEM images of graphene-covered, untreated live COS-7 cells, taken at *V*_0_=3, 2 and 5 kV, respectively. (**h**) SEM image of a graphene-covered, fixed wet COS-7 cell that was stained with 2% uranyl acetate (*V*_0_=4 kV). (**i**) SEM image of a graphene-covered, wet COS-7 cell that was fixed and membrane-extracted for preservation of the actin cytoskeleton and stained with tannic acid and uranyl acetate. *V*_0_=5 kV. (**j**) Zoom-in of **i**. (**k**) Close-up of a sparse region, and cross-section through one filament along the dotted line. Scale bars, 1 mm (**a**); 50 μm (**b**,**c**); 10 μm (**d**–**g**); 4 μm (**h**); 2 μm (**i**); 1 μm (**j**); 100 nm (**k**).

Having verified that graphene can provide a hermetic seal for cells, we moved forward to examine its applicability to electron microscopy of wet cells under high-vacuum conditions. Graphene sheets were deposited onto wet cells cultured on coverglass such that most of the coverglass surface was overlaid with graphene. Silver paint was used to contact a corner of the deposited graphene sheet to the sample holder for dissipation of electric charge during electron microscopy (Fig. 2a, ‘Ag'). The sample was then loaded into a conventional scanning electron microscope (SEM) operated under standard secondary electron mode. Normal operational vacuum (5 × 10^−7^–2 × 10^−5^ torr, depending on the particular system) was readily reached during pump down.

We first examined fixed cell samples that were briefly stained with a 0.5% uranyl acetate solution but otherwise remained fully hydrated. Under SEM, the non-covered, non-conductive parts of the sample rapidly accumulated electric charge, leading to excessively bright and unstable signals (Fig. 2a,b). Zoomed-in images (Fig. 2b) displayed limited contrast and abnormal cell morphology attributable to structural deformation under vacuum. In contrast, graphene-covered regions are characterized by stable SEM signal with no indication of charge accumulation (Fig. 2c). Graphene-covered cells can thus be imaged with good contrast over the entire field of view (Fig. 2a) and at higher magnifications (Fig. 2c,d). Cell morphology was free of visible artefacts in all cases examined, indicating good preservation of cellular structures in vacuum. For cells that were fixed and membrane-extracted for preservation of the actin cytoskeleton^1^,^2^,^31^, the obtained SEM images correlated well with conventional fluorescence images of phalloidin-labelled actin (Supplementary Fig. 2) while providing finer structural details.

We then applied the same strategy to untreated live cells. At an accelerating voltage (*V*_0_) of 3 kV, substantial contrast was obtained for the internal structure of the graphene-covered, untreated cells (Fig. 2e). Void structures with low electron density, typically 200 nm–2 μm in size, are observed in cells, likely corresponding to vesicle-like organelles that physically exclude the cytosol. Lower *V*_0_ (2 kV) led to less transparent images, but was helpful in outlining the overall cell morphology (Fig. 2f). At higher *V*_0_ (5 kV), the untreated cells became overly transparent with only the nuclei providing contrast (Fig. 2g). Previous studies using polyimide or silicon nitride membranes as imaging windows for electron microscopy of wet cells necessitate the use of high *V*_0_ (>10 kV) to penetrate the relatively thick (>∼50 nm) membranes, thus providing limited contrast on unstained animal cells^10^,^11^,^12^. As an ultrathin membrane, graphene interacts minimally with the electron beam^23^,^24^ and thus allows for cell imaging at much lower *V*_0_. The fact that graphene is an excellent thermal and electrical conductor further reduces damage by the electron beam so that the same unfixed cells can be imaged multiple times and under different conditions without noticeable structural changes (Fig. 2e-g).

Enhanced image contrast was obtained for wet samples that were suitably fixed and stained. For fixed cells that were not membrane-extracted, staining with a 2% aqueous solution of uranyl acetate revealed the structural details of the plasma membrane and mitochondria (Fig. 2h). For samples fixed and membrane-extracted for preservation of the actin cytoskeleton^1^,^2^,^31^, a two-step staining with tannic acid and uranyl acetate solutions^2^,^10^ led to excellent contrast under graphene, enabling electron microscopy of individual cytoskeletal actin filaments in hydrated samples for the first time (Fig. 2i,j and Supplementary Fig. 3). Line scans over single filaments produced cross-sectional widths of ∼14 nm (Fig. 2k), close to the known diameter of actin filaments (8 nm) and limited by the achievable resolution of the SEM systems we used. The obtained outstanding resolution and contrast are again attributed to the ultimate thinness of graphene. As a uniform, single layer of carbon atoms, graphene causes minimal electron scattering^23^,^24^ and is thus instrumental in revealing the detailed structures of the covered cells.

### Correlative super-resolution and electron microscopy

**Figure 3:**
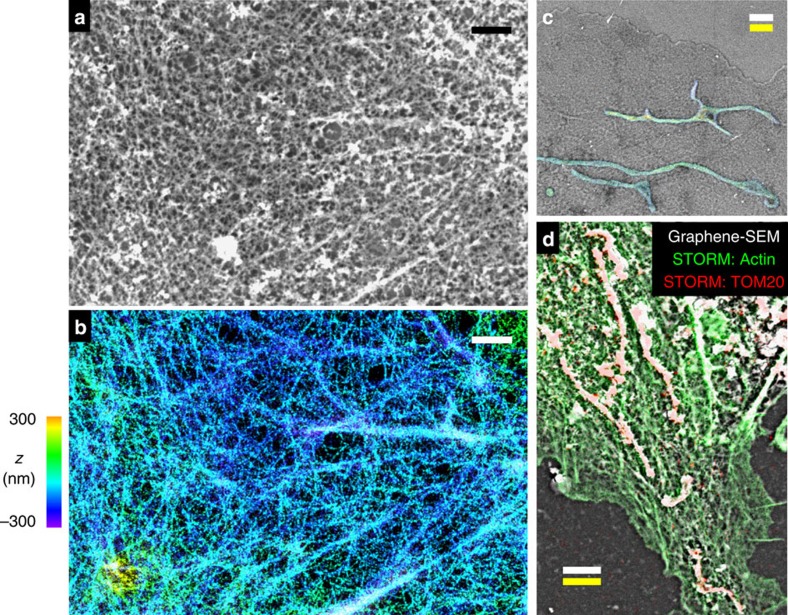
Graphene-enabled correlated super-resolution and electron microscopy of wet cells. (**a**,**b**) Correlated graphene-SEM (**a**) and 3D-STORM (**b**) images of the actin cytoskeleton in a wet, fixed and membrane-extracted COS-7 cell. (**c**) Correlated and overlaid graphene-SEM and 3D-STORM images of a wet, fixed COS-7 cell that was not membrane extracted (Fig. 2h). For STORM, the sample was immunolabelled for TOM20, a mitochondrial outer-membrane marker. (**d)** Correlated and overlaid two-colour STORM (green for actin; red for TOM20) and graphene-SEM (white) images for another membrane-extracted fixed cell. Colour scale in **b** is used to indicate height (*z*) in **b**,**c**. Scale bars, 1 μm (**a**,**b**); 2 μm (**c**,**d**). White and yellow scale bars in **c**,**d** correspond to scales obtained from graphene-SEM and STORM, respectively.

Owing to its compatibility with wet samples on standard coverglass, our method can be readily extended to allow for correlative^4^,^5^,^6^ super-resolution and electron microscopy. Here we used three-dimensional stochastic optical reconstruction microscopy (3D-STORM)^32^,^33^ to first resolve actin filaments in fixed wet cells on coverglass^31^,^32^,^33^, and then uranyl stained the sample and applied graphene for correlated SEM imaging. Comparison of the 3D-STORM and graphene-SEM images shows good correspondence of actin ultrastructure, enabling correlation of individual actin filaments between super-resolution and electron microscopy images (Fig. 3 a,b and Supplementary Figs 4,5 and 11). Excellent correlative STORM/graphene-SEM results were also obtained for the cell membrane in unstained cells (Supplementary Fig. 6) and for mitochondria in stained cells (Fig. 3c and Supplementary Figs 7 and 8). Figure 3d further shows a case in which actin filaments and mitochondria are both visualized in the same sample. Two-colour STORM images show good correlation with SEM for both structures. Furthermore, good agreement is obtained between the scale bars obtained from STORM and SEM measurements in all cases, confirming preservation of volume and size of wet cells in vacuum (Fig. 3 and Supplementary Figs 5–8). Taken together, these results indicate preservation of fine structural details in graphene-covered wet samples.

## Discussion

A considerable obstacle in electron microscopy of cell samples has been achieving proper preservation of fine cellular structure during the conventionally required sample dehydration procedures. Both air- and freeze-drying lead to major distortions (for example, Supplementary Figs 9–11)^1^,^2^. Dehydration through a graded series of organic solvents followed by critical-point drying and platinum/carbon deposition has been successful, but is technically challenging and time consuming^2^. By taking full advantage of the extraordinary properties of graphene as the thinnest membrane that is impermeable and conductive, our approach allows for direct electron microscopy of wet cells through a simple, one-step sample preparation. No special substrate, device or equipment is involved, and good contrast and resolution are achieved with conventional SEM. Its ready application to cells cultured on standard coverglass further permits facile correlation with super-resolution microscopy for multiple targets in unstained and stained cells. Our approach thus opens up new ways to examine biological samples at the nanoscale in their native, hydrated state.

## Methods

### Cell culture and immunofluorescence labelling

Mammalian cells (BS-C-1, COS-7, HeLa; American Type Culture Collection) were cultured on common glass coverslips (typically 12 mm diameter) following standard tissue culture protocols. For live cell experiments (Fig. 2e–g), cells were left untreated before the application of graphene. For correlated STORM and graphene-SEM of unstained cells (Supplementary Fig. 6), live cells were labelled with a CM-DiI cell membrane-labelling solution (Invitrogen V-22888) at 20 μM in DMEM for 5 min, and then fixed by 4% paraformaldehyde for 10 min. For experiments aimed at visualizing the actin cytoskeleton (Figs 2a–d, i–k, and 3 a,b,d, and Supplementary Figs 2–5 and 10c), cells were initially fixed and extracted for 1 min with a solution of 0.3% (v/v) glutaraldehyde and 0.25% (v/v) Triton X-100 in cytoskeleton buffer (CB, 10 mM MES, pH 6.1, 150 mM NaCl, 5 mM EGTA, 5 mM glucose and 5 mM MgCl_2_), and then post-fixed for 20 min in 2% (v/v) glutaraldehyde in CB^1^,^2^,^31^. For other fixed-cell experiments, cells were fixed in 3% formaldehyde and 0.1% glutaraldehyde in phosphate buffered saline (PBS) for ∼10 min. For immunofluorescence labelling, cells were first blocked with a solution of 3% bovine serum albumin and 0.1% Triton X-100 in PBS, and then stained with corresponding primary and secondary antibodies. Primary antibodies used were mouse anti-tubulin (Sigma, T5201; 1:400) for labelling of microtubules and rabbit anti-Tom20 (Santa Cruz, sc11415; 1:200) for labelling of mitochondria. For single-colour and two-colour STORM imaging of mitochondria, AF647-conjugated and Cy3B-conjugated^34^ secondary antibodies (at 5 μg ml^−1^) were used to label Tom20, respectively. For fluorescent labelling of actin filaments, samples were incubated^31^ with AF488-conjugated phalloidin (Invitrogen, A12379; for Fig. 1c), AF555-conjugated phalloidin (Invitrogen, A34055; for Fig. 1f) or AF647-conjugated phalloidin (Invitrogen A22287; for all other data) at a concentration of ∼0.4 μM.

### Staining for electron microscopy

For data presented in Fig. 2a–d and Supplementary Fig. 2, fixed and membrane-extracted cells were stained with 0.5% uranyl acetate (SPI 02624) in water for 5–10 min, washed three times with water and kept in water before graphene deposition. For imaging of mitochondria and plasma membrane (Figs 2h and 3c and Supplementary Figs 7, 8, and 10a), fixed cells were stained with 2% uranyl acetate in water for 1 h. For improved contrast of the actin cytoskeleton (Figs 2i–k, and 3a,d, and Supplementary Figs 3 and 4), samples were treated with 5% tannic acid (Sigma, 403040) in water for 5 min, followed by a solution of 2% uranyl acetate in water for 2 h. Samples were thoroughly washed with water and kept in water before graphene deposition.

### Graphene deposition

CVD graphene on copper foil^35^ were grown at Cornell NanoScale Science and Technology Facility or purchased from Graphene Supermarket. Similar results were obtained using graphene from the two sources. The CVD graphene on copper foil was spin coated with a ∼150-nm layer of polymethyl methacrylate (PMMA), and cut into pieces slightly smaller than the size of the coverslip. After the copper was removed in 10% ferric chloride, the graphene-PMMA stack was transferred to a fresh water bath so it floated on the water surface. Water bath transfer was repeated three times to remove traces of ferric chloride. To cover cells with graphene, the hydrated coverslip containing the cells was used to scoop up the graphene-PMMA stack floating on water. The stack was allowed to adhere to the sample for ∼10 min in air. To remove PMMA, the sample was dipped in anisole or acetone for 2 min, and rinsed off briefly in isopropyl alcohol. Deposited graphene was identified in bright-field microscopy as a continuous, slightly darkened film (Fig. 1b), likely due to the known absorption of graphene to 2.3% of white light^36^. Near 100% yield was achieved. Quality of graphene was evaluated via Raman spectroscopy. Raman spectra were recorded with a Renishaw InVia micro-Raman system using a 488-nm laser and a 2,400 lines per mm grating. A confocal microscope with a × 50 objective lens was used to record spectra at a spatial resolution of ∼2 μm. Raman spectroscopy confirmed that the graphene used in this study was high-quality monolayer (Fig. 1d and Supplementary Fig. 12)^30^. We have also found that small amounts of bilayers do not notably affect our results, but low-quality graphene with excessive bilayers and defects is not optimal for obtaining the best results with our method (Supplementary Fig. 12).

### SEM imaging

The graphene-covered coverslip was mounted on a standard metallic sample mount with carbon tape, and a small amount of silver colloid paint (Ted Pella 16031) was used to create a conductive bridge between graphene and the sample mount. SEM imaging was performed under standard secondary electron mode on a FEI Quanta 3D FEG system or a JEOL JSM-6340F system. Normal operational vacuum (5 × 10^−7^–2 × 10^−5^ torr) was readily reached during pump down. Calibration of magnification was verified with a replica of a 2,160 lines per mm waffle-pattern diffraction grating (Ted Pella 604-A).

### Correlative STORM/Graphene-SEM imaging

To facilitate location of the same cells under STORM and SEM, a diamond scribe was used to make a scratch mark (for example, ∼1 mm^2^ triangular) at the centre of the coverslip, which was readily identifiable both under optical microscope and in SEM under the coverage of graphene. 3D-STORM imaging^32^,^33^ was first performed on a homebuilt setup based on a Nikon Eclipse Ti-E inverted optical microscope using an oil immersion objective (Nikon CFI Plan Apochromat λ × 100, numerical aperture=1.45). Lasers at 647 nm (MPB Communications), 560 nm (MPB Communications) and 405 nm (Coherent) were coupled into an optical fibre after an acousto-optic tunable filter and then introduced into the sample through the back focal plane of the microscope. Using a translation stage, the laser beams were shifted towards the edge of the objective so that emerging light reached the sample at incidence angles slightly smaller than the critical angle of the glass–water interface. Continuous illumination of 647-nm laser (∼2 kW cm^−2^; for STORM of AF647) or 560-nm laser (∼2 kW cm^−2^; for STORM of Cy3B and CM-DiI) was used to excite fluorescence from labelled dye molecules and switch them into the dark state. Concurrent illumination of the 405-nm laser was used to reactivate the fluorophores to the emitting state. The power of the 405-nm laser (typical range 0–1 W cm^−2^) was adjusted during image acquisition so that at any given instant, only a small, optically resolvable fraction of the fluorophores in the sample were in the emitting state. For 3D-STORM imaging, a cylindrical lens was inserted into the imaging path so that images of single molecules were elongated in *x* and *y* for molecules on the proximal and distal sides of the focal plane (relative to the objective), respectively^33^. Imaging buffer used was Tris-Cl containing 100 mM cysteamine, 5% glucose, 0.8 mg ml^−1^ glucose oxidase and 40 μg ml^−1^ catalase. After STORM imaging, the coverslip was stored in PBS before processing for graphene-based SEM imaging (as described above). To align the obtained STORM and SEM images, the STORM image was mapped to the coordinate system of the SEM image through a two-dimensional affine spatial transformation (MATLAB) on the basis of corresponding features (control points). About 20 control points were selected in each data set for inferring an averaged, global, affine transformation matrix.

## Additional information

**How to cite this article**: Wojcik, M. *et al*. Graphene-enabled electron microscopy and correlated super-resolution microscopy of wet cells. *Nat. Commun.* 6:7384 doi: 10.1038/ncomms8384 (2015).

## Supplementary Material

Supplementary Information Supplementary Figures 1-12.
