# Supplementary material for: Graphene-enabled electron microscopy and correlated super-resolution microscopy of wet cells
Source: Nat Commun. 2015 Jun 11;6:7384. doi: 10.1038/ncomms8384 (PMC4490578; doi:10.1038/ncomms8384)
Supplement: Supplementary Information — Supplementary Figures 1-12. [file ncomms8384-s1.pdf]

Graphene-enabled electron microscopy and correlated super-resolution microscopy of wet cells

Michal Wojcik[1†], Margaret Hauser[1†], Wan Li[1], Seonah Moon[1], Ke Xu[1,2]*

[1]Department of Chemistry, University of California, Berkeley, California 94720, USA.

[2]Life Sciences Division, Lawrence Berkeley National Laboratory, Berkeley, California 94720, USA

†These authors contributed equally to this work.

*Correspondence to: xuk@berkeley.edu.

Supplementary Information

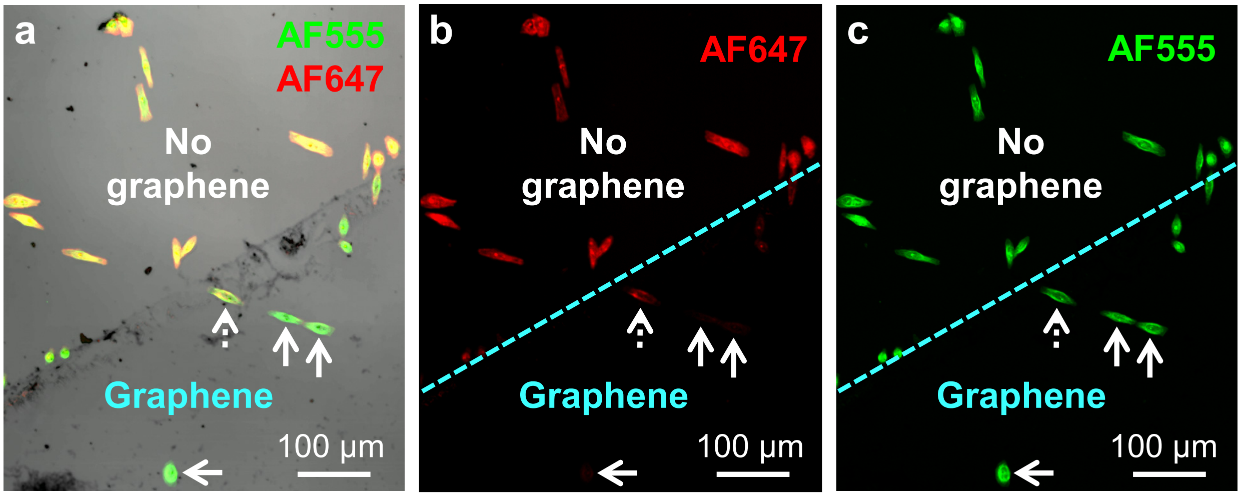

Supplementary Figure 1. Graphene insulates cells from the external environment over long periods. a, Combined fluorescence and bright-field microscopy images of fixed HeLa cells that were pre-labeled with Alexa Fluor 555 (AF555) for tubulin (green), partly covered with graphene, and incubated with AF647-phalloidin (red), a potent stain for cytoskeletal actin, for 16 h. **b,** The AF647 channel. **c,** The AF555 channel. Cells not covered by graphene were strongly labeled by AF647, whereas no noticeable labeling was observed for graphene-covered cells (solid arrows). As a control, the pre-labeled AF555 appeared equally strong for all cells. Cells close to the graphene edge are lightly stained by AF647 (e.g., dashed arrow), suggesting that leakage may gradually occur from the graphene edge over time.

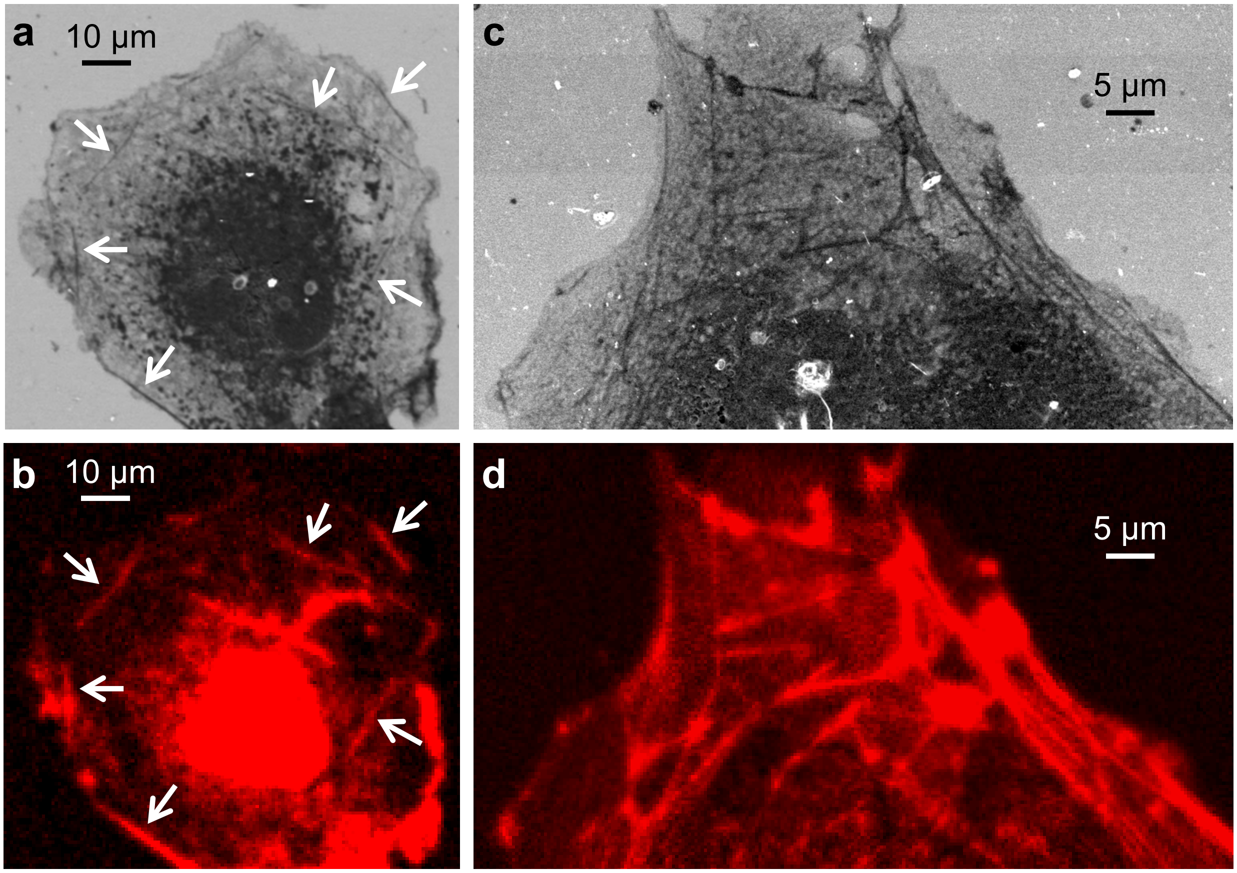

Supplementary Figure 2. Correlated SEM and conventional fluorescence microscopy for graphene-covered, membrane-extracted wet cells. **a**, Graphene-SEM image, corresponding to Fig. 2d in main text. **b**, Corresponding fluorescence microscopy of phalloidin-labeled actin. Arrows point to stress fibers that are visualized in both images. **c,d**, Graphene-SEM (**c**) and correlated fluorescence microscopy of phalloidin-labeled actin (**d**) for another cell.

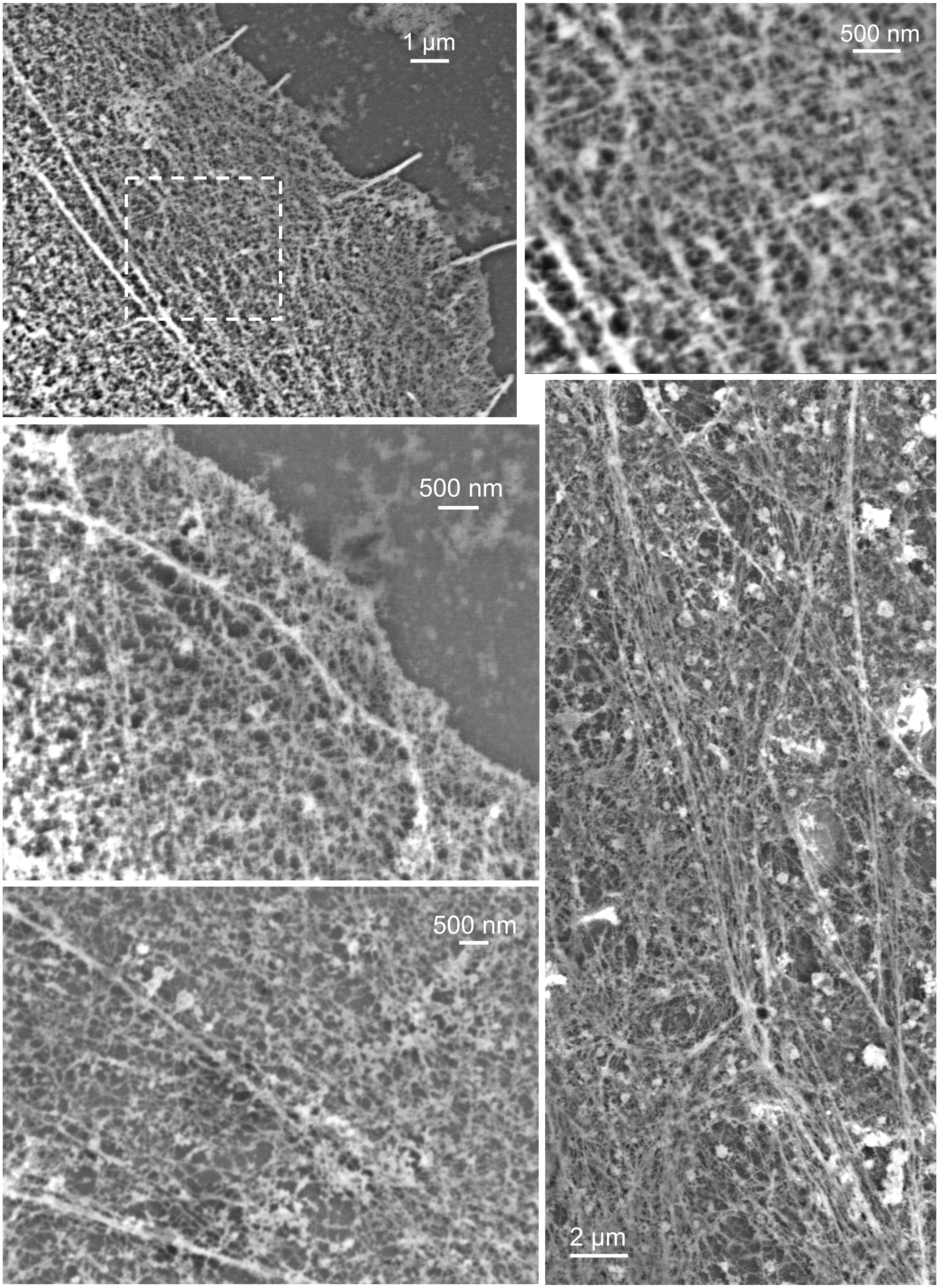

Supplementary Figure 3. Additional graphene-SEM images for the actin cytoskeleton in wet cells.

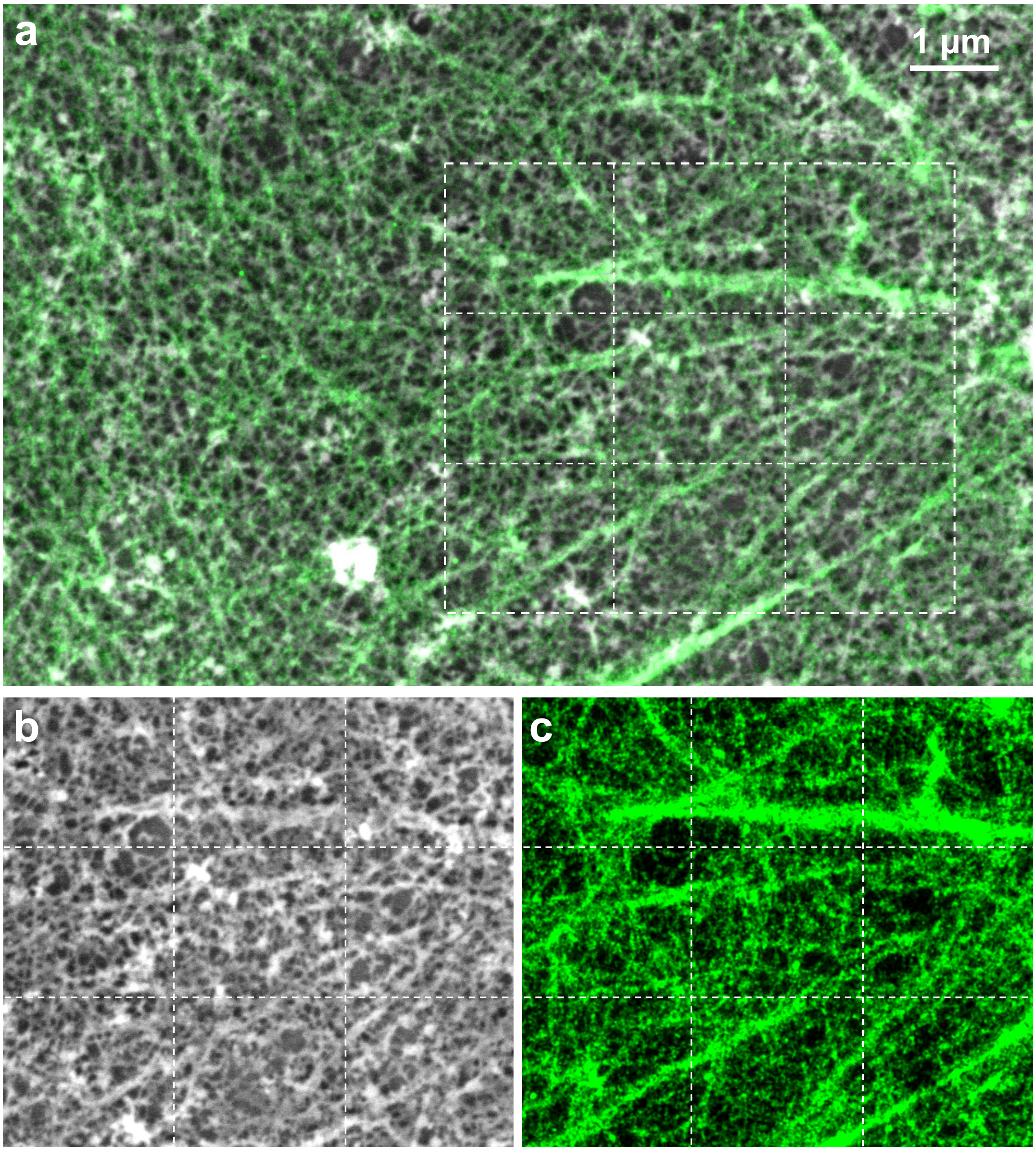

Supplementary Figure 4. Comparison of the correlated graphene-SEM and STORM images. a, Magnified and overlaid graphene-SEM (white) and STORM (green) images corresponding to Fig. 3ab. **b,c**, Graphene-SEM (**b**) and STORM (**c**) images corresponding to the boxed area in **a**. Grid lines are added to aid comparison of the images. Good correlation is generally observed. Local differences in structural details may be partly attributed to insufficient fluorescence labeling of the actin filaments in STORM and non-actin structures that only show up in SEM.

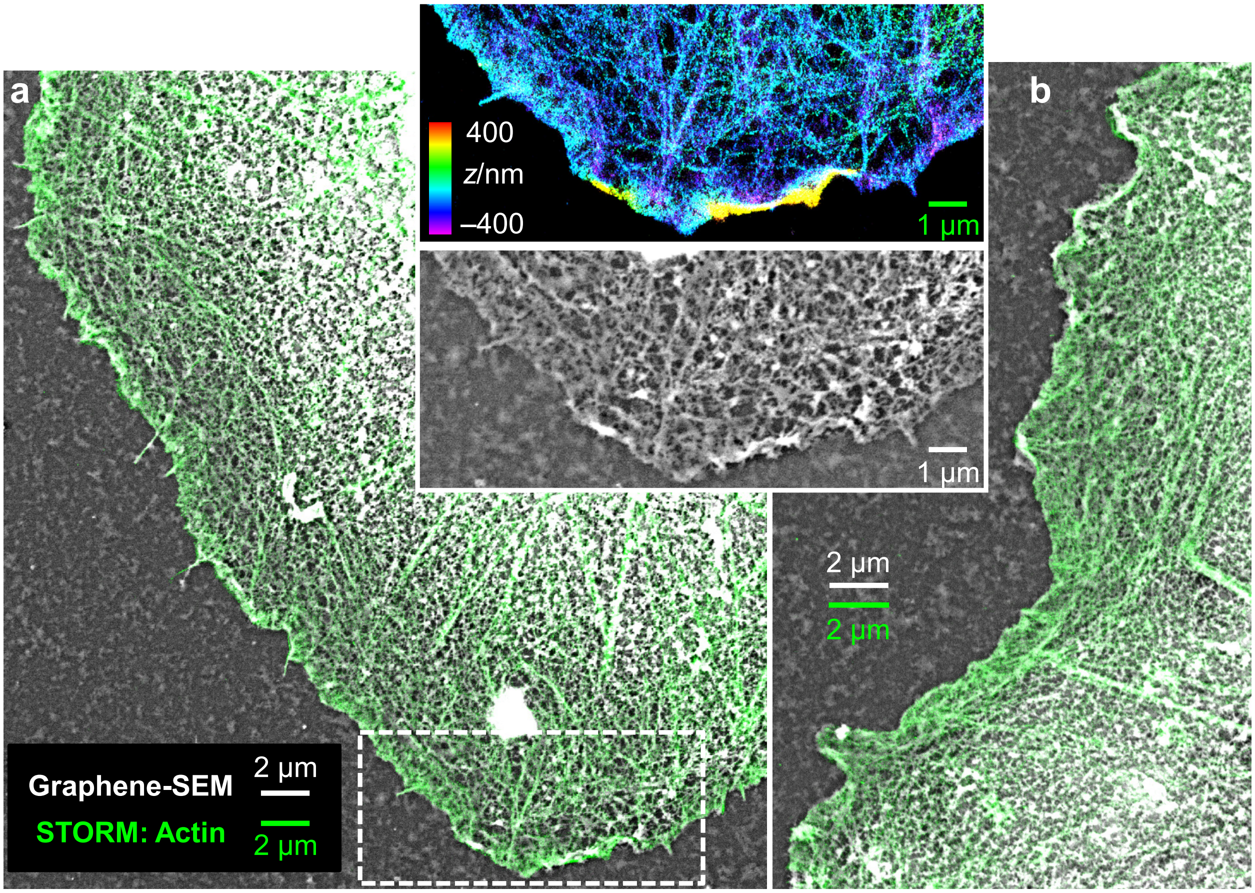

Supplementary Figure 5. Additional correlated STORM and graphene-SEM results for the actin cytoskeleton in wet cells. a,b, Correlated STORM (green) and graphene-SEM (white) images of fixed, wet COS7 cells. **Insets:** zoom-in of the 3D-STORM image (top) and Graphene-SEM image (bottom) for the boxed area in **a**. Color scale is used to indicate height (*z*) in the 3D-STORM image. White and green scale bars correspond to scales obtained from graphene-SEM and STORM, respectively.

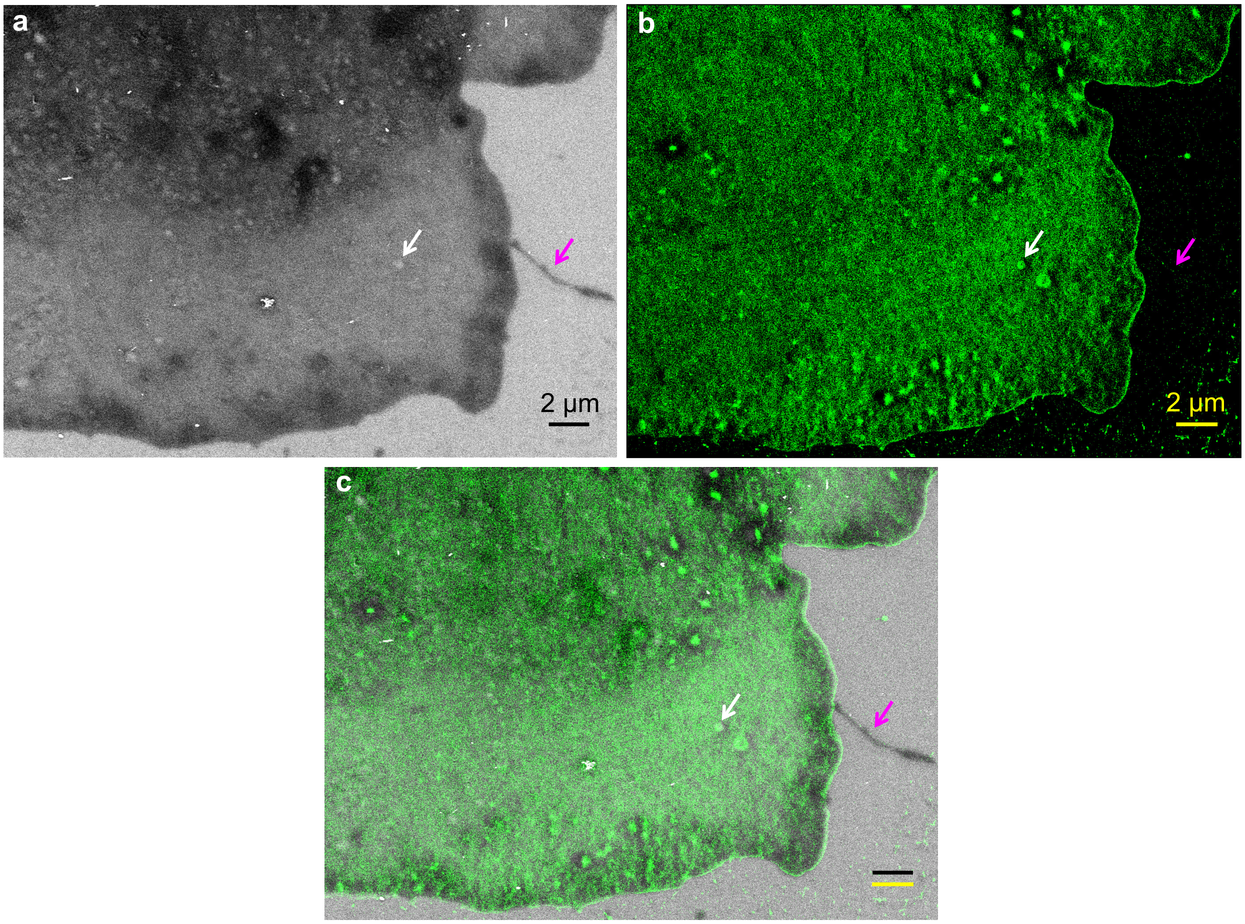

Supplementary Figure 6. Correlated STORM and graphene-SEM results for an unstained wet cell. Live COS-7 cells were labeled with a DiI cell membrane-labeling solution (Invitrogen V-22885) at 20 μM in DMEM for 5 min, and then fixed by 4% paraformaldehyde. **a**, Graphene-SEM image. V_0 = 2 kV (for best contrast of cell shape). **b**, Correlated STORM super-resolution image of the labeled DiI, imaged with a 560 nm laser. **c**, Overlaid image. Black and yellow scale bars correspond to scales obtained from graphene-SEM and STORM, respectively. White arrow points to a vesicle that is visualized in both the graphene-SEM and STORM images: This is likely due to the local internalization of the cell membrane (e.g., endocytosis) during dye labeling of the live cell. Meanwhile, many other vesicles are observed in the graphene-SEM image but not in the STORM image: these are likely internal vesicles that are not labeled by the DiI solution. Occasionally observed clusters in the STORM image are attributed to undissolved DiI aggregates. Magenta arrow points to a structure that is visualized in graphene-SEM but barely visible in STORM due to the low labeling of DiI therein.

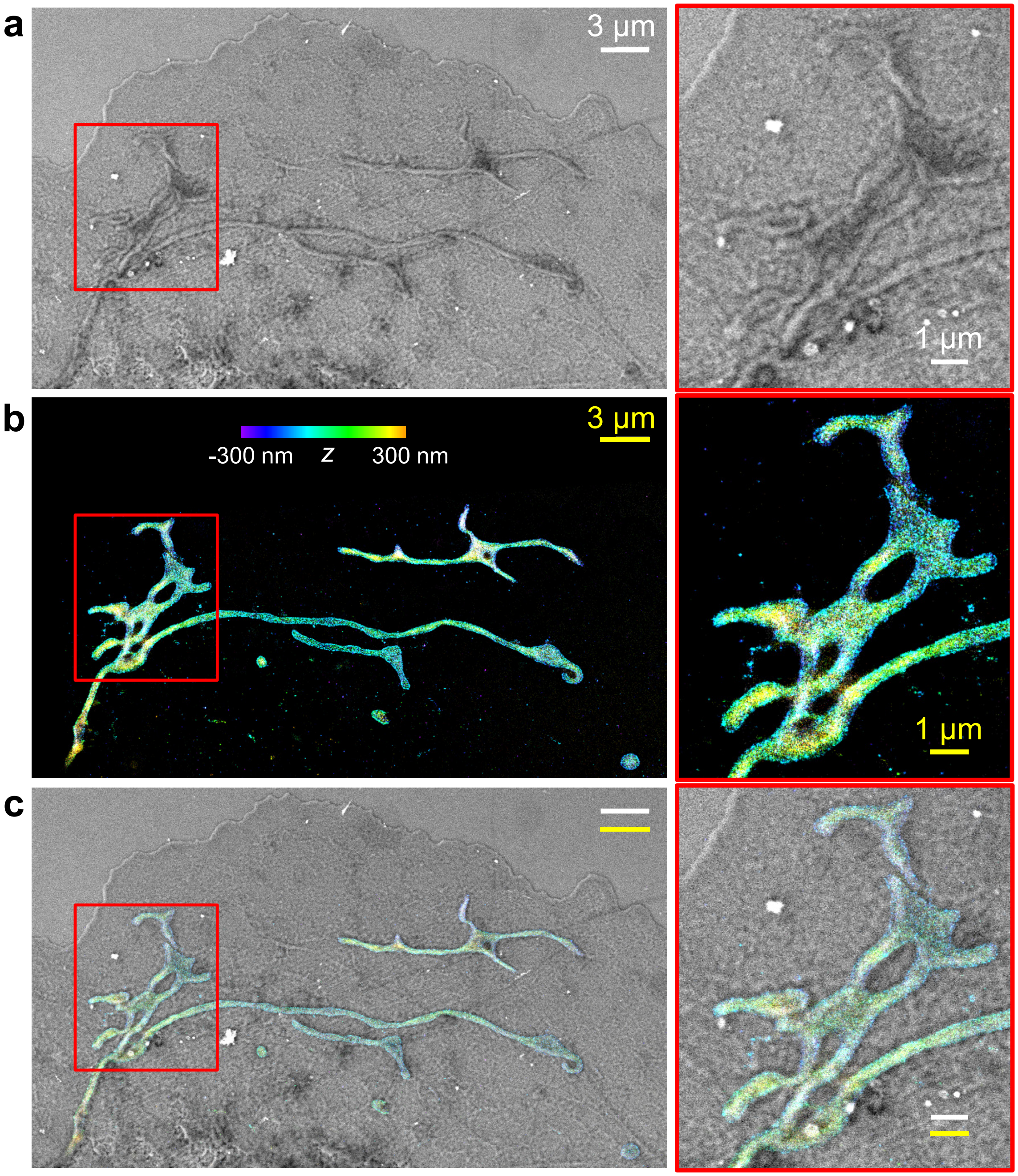

Supplementary Figure 7. Details of correlated electron microscopy and super-resolution microscopy of mitochondria in the wet cell presented in Figs. 2h and 3c. a, Graphene-SEM images. **b**, Correlated 3D-STORM super-resolution images of TOM20, a mitochondrial outer-membrane marker. **c**, Overlaid images. Color scale in **b** is used to indicate height (*z*) in the 3D-STORM images. White and yellow scale bars correspond to scales obtained from graphene-SEM and STORM, respectively.

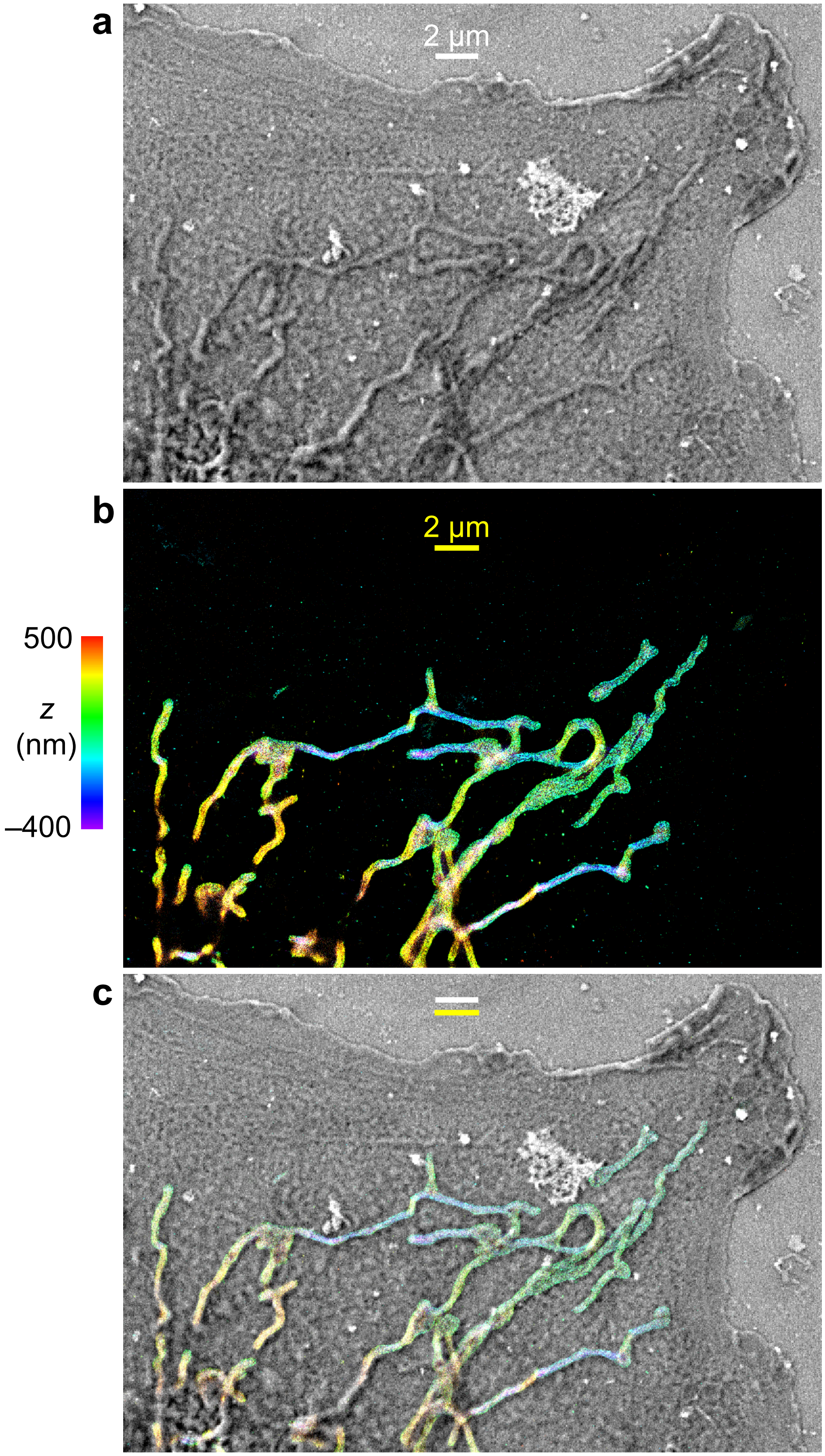

Supplementary Figure 8. Correlated STORM and graphene-SEM images of mitochondria in another wet cell. **a**, Graphene-SEM image. **b**, Correlated 3D-STORM super-resolution image of TOM20. Color scale in **b** is used to indicate height (*z*). **c**, Overlaid image.

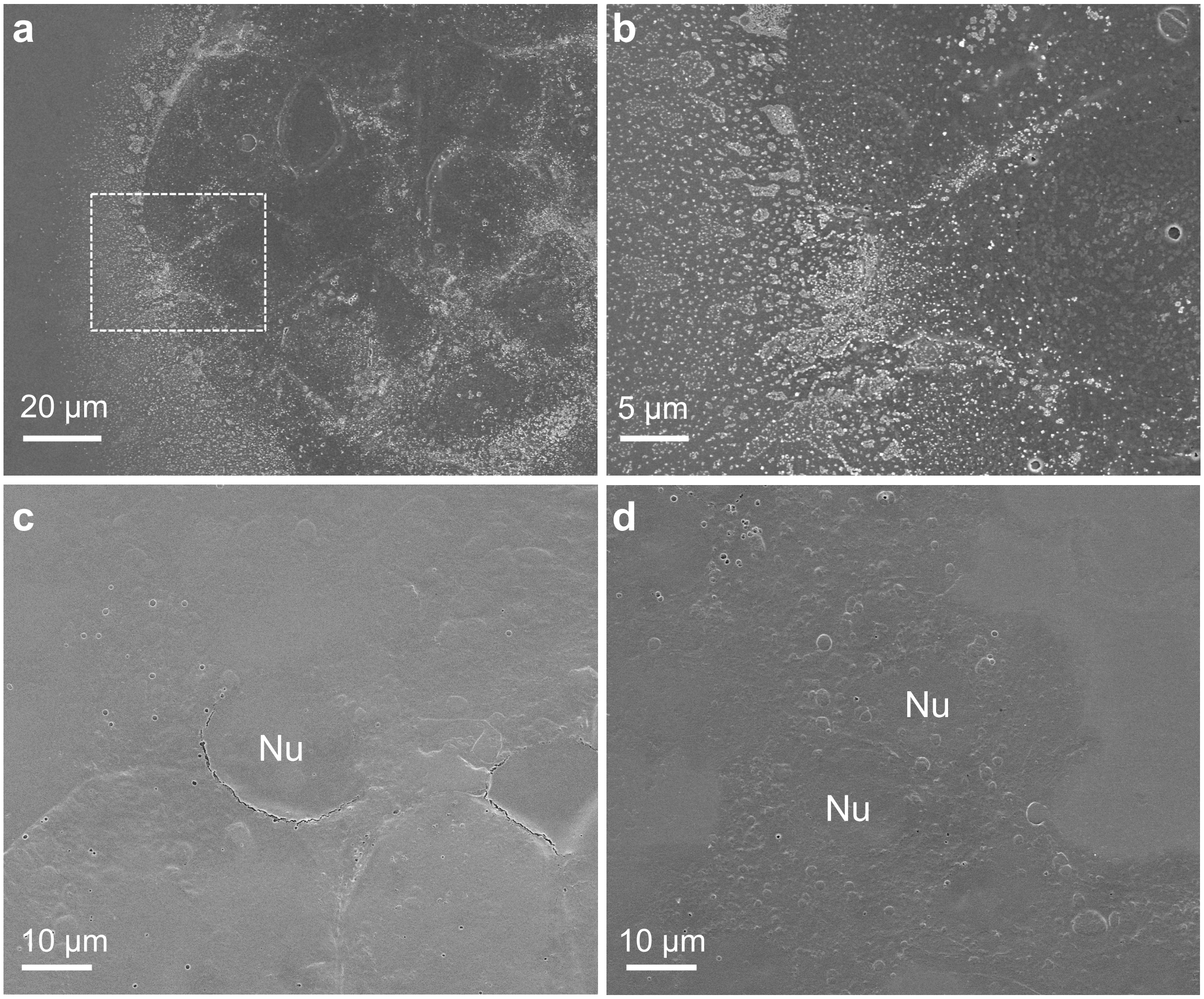

Supplementary Figure 9. SEM results on untreated and lightly fixed cells that were dehydrated. To overcome surface charging, samples were sputter-coated with a conductive, 10-nm Au film before imaging. **a**, Untreated live cells (the same conditions as Fig. 2e-g) dehydrated in vacuum. **b**, Zoom-in of the boxed area in **a**. **c**, Untreated live cells slowly dried in air. **d**, Cells were fixed with 4% paraformaldehyde (the same conditions as Supplementary Fig. 6) and then slowly dried in air.

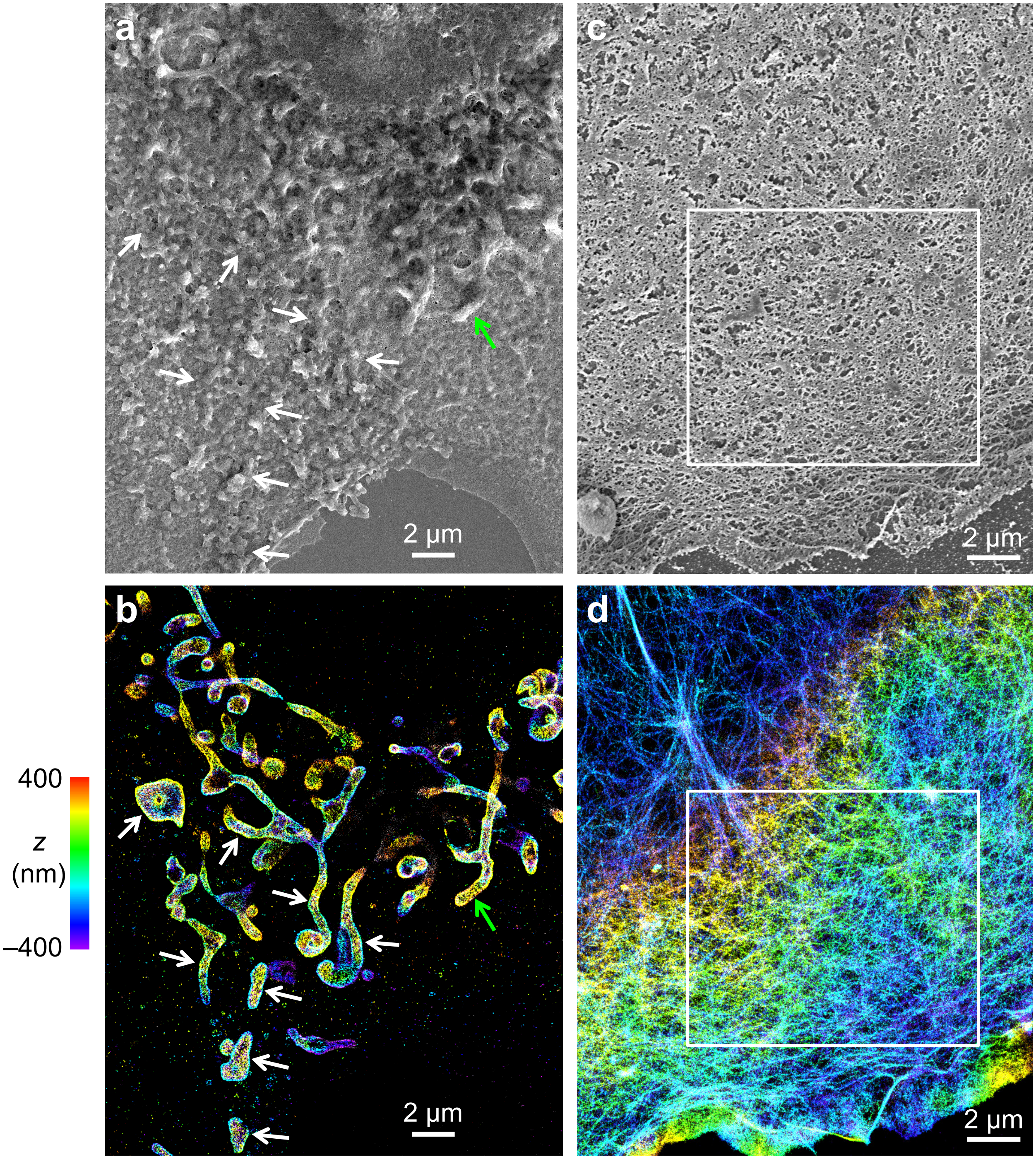

Supplementary Figure 10. Comparison of STORM and EM images for fixed and stained COS7 cells that were slowly dried in air. Samples were sputter-coated with a conductive, 10 nm Au film before EM. **a**, SEM image of a sample that was optimized for the visualization of mitochondria (the same conditions as Supplementary Figs. 7 & 8). **b**, 3D-STORM image of TOM20, taken before sample was dried. Only the feature pointed to by the green arrow showed correspondence in **a** and **b**, whereas most other mitochondria structures are significantly distorted in the dried cell (white arrows), precluding meaningful correlation with the STORM image. **c**, SEM image of a sample that was optimized for the visualization of the actin cytoskeleton (the same conditions as Fig. 2ij, Fig. 3 and Supplementary Figs. 3-5). **d**, Correlated 3D-STORM image of actin, taken before sample drying. Dried actin networks collapsed and coalesced into a two-dimensional film, reflecting a common issue in the preparation of the actin cytoskeleton for EM.

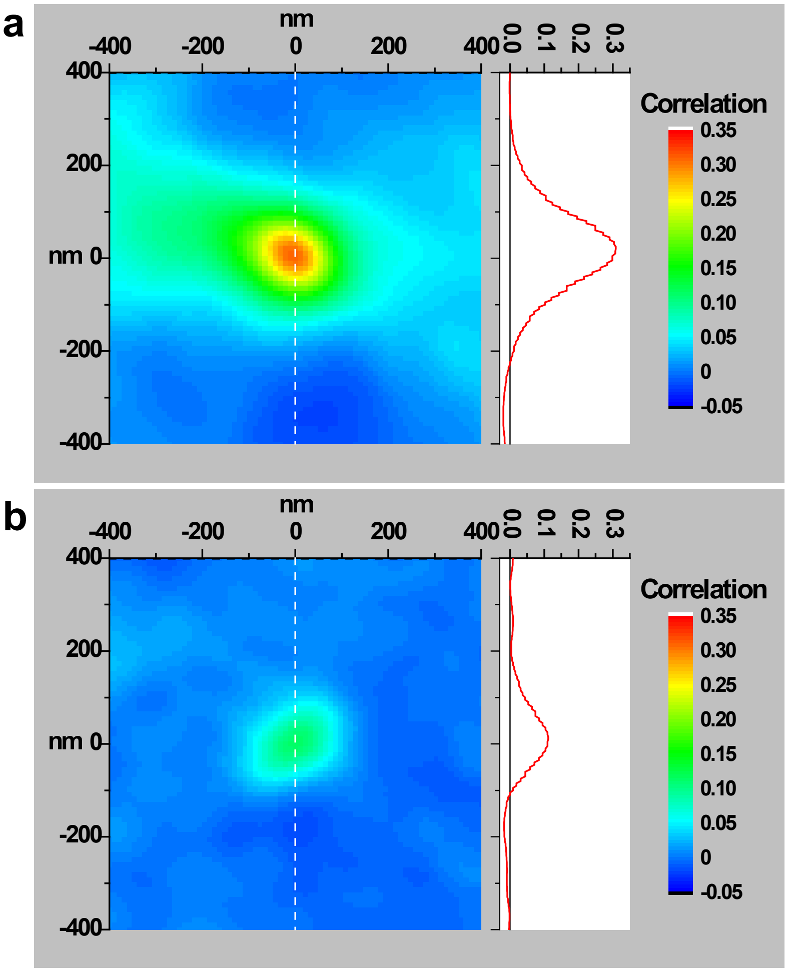

Supplementary Figure 11. Calculation of the normalized cross-correlation of correlated STORM and EM images based on pixel intensity, as a preliminary attempt to evaluate the correspondence between STORM and EM images. a, Calculated for a graphene-encased sample (Fig. 3a and Fig. 3b). A maximal cross-correlation coefficient of 0.31 is obtained, which is reasonably good considering the very different contrast mechanisms of STORM and EM. **b**, Calculated for a control sample without graphene (boxed areas in Supplementary Fig. 10d and Supplementary Fig. 10e). A much lower cross-correlations coefficient (0.11) is obtained due to distortion of the actin network upon dehydration.

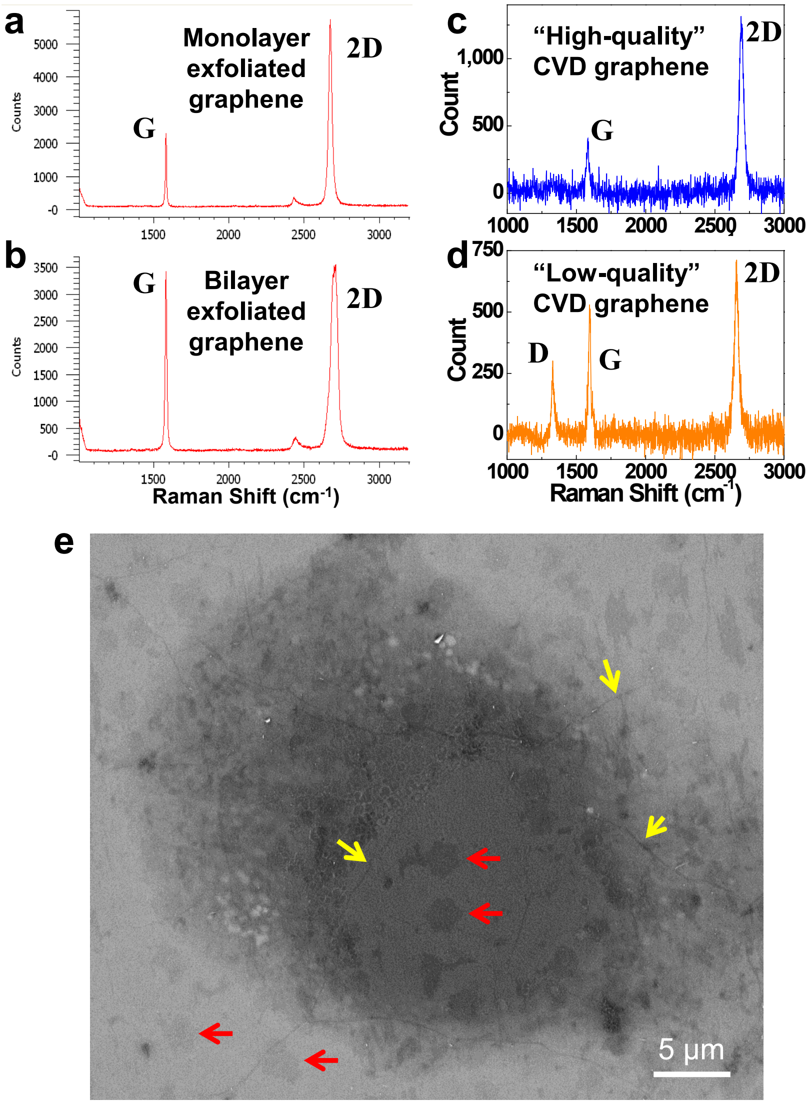

Supplementary Figure 12. Quality of graphene. a,b, Reference Raman spectroscopy of exfoliated, pristine monolayer (**a**) and bilayer (**b**) graphene on an oxidized silicon wafer. Monolayer graphene is characterized by a 2D peak that is significantly higher than the G peak, whereas bilayer graphene is characterized by a 2D peak that is similar in height when compared to the G peak. **c**, Raman spectroscopy for the CVD graphene (on coverglass) used in this study. Background from the coverglass has been subtracted (Fig. 1d). The observed Raman spectrum is comparable to that of the pristine monolayer graphene (**a**). **d**, Raman spectroscopy of low-quality CVD graphene with bilayers (decreased 2D peak to G peak ratio) and other defects (appearance of additional "D" peak). **e**, SEM of a sample (untreated BS-C-1 cell) that was prepared with graphene of particularly low quality. V_0 = 3 kV. Red arrows point to bilayer islands (often characterized by 120° corners). Yellow arrows point to wrinkle defects.
